# Epileptic Seizure Detection on an Ultra-Low-Power Embedded RISC-V Processor Using a Convolutional Neural Network

**DOI:** 10.3390/bios11070203

**Published:** 2021-06-23

**Authors:** Andreas Bahr, Matthias Schneider, Maria Avitha Francis, Hendrik M. Lehmann, Igor Barg, Anna-Sophia Buschhoff, Peer Wulff, Thomas Strunskus, Franz Faupel

**Affiliations:** 1 Sensor System Electronics, Institute of Electrical Engineering and Information Technology, Kiel University, 24143 Kiel, Germany; matthia-s@gmx.de (M.S.); maria.avitha.francis@iis.fraunhofer.de (M.A.F.); 2 CMOS Design, Technical University Braunschweig, 38106 Braunschweig, Germany; Hendrik.Lehmann@infineon.com; 3 Multicomponent Materials, Institute for Material Science, Kiel University, 24143 Kiel, Germany; igba@tf.uni-kiel.de (I.B.); ts@tf.uni-kiel.de (T.S.); ff@tf.uni-kiel.de (F.F.); 4 Institute of Physiology, Kiel University, 24118 Kiel, Germany; a.buschhoff@physiologie.uni-kiel.de (A.-S.B.); p.wulff@physiologie.uni-kiel.de (P.W.)

**Keywords:** convolutional neural network, EEG, epileptic seizure detection, RISC-V, ultra-low-power

## Abstract

The treatment of refractory epilepsy via closed-loop implantable devices that act on seizures either by drug release or electrostimulation is a highly attractive option. For such implantable medical devices, efficient and low energy consumption, small size, and efficient processing architectures are essential. To meet these requirements, epileptic seizure detection by analysis and classification of brain signals with a convolutional neural network (CNN) is an attractive approach. This work presents a CNN for epileptic seizure detection capable of running on an ultra-low-power microprocessor. The CNN is implemented and optimized in MATLAB. In addition, the CNN is also implemented on a GAP8 microprocessor with RISC-V architecture. The training, optimization, and evaluation of the proposed CNN are based on the CHB-MIT dataset. The CNN reaches a median sensitivity of 90% and a very high specificity over 99% corresponding to a median false positive rate of 6.8 s per hour. After implementation of the CNN on the microcontroller, a sensitivity of 85% is reached. The classification of 1 s of EEG data takes t=35 ms and consumes an average power of P≈140 μW. The proposed detector outperforms related approaches in terms of power consumption by a factor of 6. The universal applicability of the proposed CNN based detector is verified with recording of epileptic rats. This results enable the design of future medical devices for epilepsy treatment.

## 1. Introduction

With about 1% of the population affected, epilepsy is one of the most common neurological diseases globally [1]. Epilepsy requires ongoing medical attention and is associated with a decrease in the patients’ quality of life and higher mortality rates [2,3]. Every year in the USA alone, the direct medical expenses including lost or reduced earnings associated with epilepsy are estimated to be $15.5 billion [4]. Despite ongoing research and development of new AEDs [5,6], the most common treatment in form of systemic administration of anti-epileptic drugs (AEDs) does not achieve sufficient long-term seizure suppression in ~30% of the patients. Therefore, alternative treatment methods to refractory epilepsy such as intracranial drug delivery [7] or neurostimulation [8] have been suggested. The pinnacle of development would be an implantable closed-loop system for on-demand intervention during ictal periods, which have to be identified sufficiently fast through an automated seizure classification system.

Since the beginning of research on automated classification of epileptic seizures in the 1970s, several algorithms to detect seizures have been developed [9,10]. The challenge in classical approaches of seizure detection is developing a model that is capable of dealing with the changing characteristics of seizures within the same subject. The different approaches rely on feature extraction coupled with a classification strategy. These features are for example wavelet-based filters, frequency band and spectral analysis, the slope, height, and duration characteristics of a seizure or cross-channel correlations [10]. Recently, with a view at hardware efficiency in order to enable implantable systems, convolutional neural networks have been analyzed for seizure detection algorithms. Lawhern et al. [11] showed with EEGNet that state-of-the-art seizure classification and interpretation is possible with a compact convolutional neural network. A CNN optimized for ultra-low power requirements was introduced in [12]. The detector, called SeizureNet, reaches a median sensitivity of 0.96 long-term for invasive intracranial EEG recordings. The efficiency of epileptic seizure prediction based on deep learning is analyzed and compared in [13,14].

Biomedical implantable and wearable devices are usually limited by size and energy restrictions. To meet the devices’ high energy efficiency requirements and form factor budget, many functions are incorporated into the device by application-specific integrated circuits (ASICs) [15,16]. Applying this concept, several applications have been developed successfully, going far beyond standard applications like pacemakers and hearing aids. A very small and lightweight bioelectric recording system for flying insects has been shown in [17]. An implantable cortical microstimulator for Brain–Computer Interfaces was realized in [18]. Benabi et al. [19] demonstrated that a tetraplegic patient can control an exoskeleton by an implanted epidural wireless brain–machine interface. In [15,16], it has been shown that an electronic system can be miniaturized to such an extent that even neural recording from neonatal mice to monitor growing processes of the brain can be feasible. The integration of digital logic into systems with low-power microcontrollers based on RISC-V architecture is promising to further advance this field of research. The RISC-V architecture is based on the reduced instruction set computer principles introduced by the University of Berkeley, California. It is available under an open source license and thus, unlike most other microcontrollers, free to use.

In this paper, an epileptic seizure detector suitable for ultra-low-power RISC-V embedded processors and based on a CNN is presented. The implementation, training, and verification of the CNN is performed in MATLAB using the open source CHB-MIT dataset. The main requirement for the detection algorithm is the feasibility for an ultra-low-power hardware implementation. At the same time the detection algorithm has to achieve state-of-the-art detection performance. Low-power and low-complexity architectures call for dimensionality reduction and small memory usage. A multi-channel EEG is a high dimensional dataset and memory usage is thus a challenge. In this work, a CNN fulfilling the low-power and low-complexity requirements is presented. It consists of only a few layers and a manageable number of weights, thus fulfilling low memory requirements.

The paper is structured as follows. Section 2 presents the dataset and Section 3 the microcontroller hardware. In Section 4, the CNN architecture and its training and implementation in Matlab are presented. The implementation of the classifier on a RISC-V-based embedded microcontroller is presented in Section 4.4. The transferability of the proposed detector and its functionality is cross-validated and proven with EEG data from a rodent absence epilepsy model (Genetic Absence Epilepsy Rats from Strasbourg (GAERS)) in Section 4.5. In Section 4.6, the capability of the developed classifier to predict epileptic seizures is demonstrated. Section 5 presents the measurements and results of the performane of the detector for each of the presented chapters. The performance of the classifier is compared with similar state-of-the-art approaches in Section 6.

## 2. Dataset

In this work the open source CHB-MIT dataset, collected at the Children’s Hospital Boston, is used [20,21]. It contains continuous scalp EEG recordings from 24 children with intractable seizures, which have been labeled by medical professionals. The type of epilepsy is not specified in [20,21]. However, this does not limit the performance of classification, as our network is independent of specific epilepsy types, but instead is trained patient specifically. The EEG sampling frequency for all patients was 256Hz. Most recordings contain 23 channels with EEG signals. For electrode positioning the international 10–20 system of EEG electrode positions and nomenclature is used. Overall, this dataset contains approximately 865 h of EEG signals with 198 seizures that usually last several seconds.

In addition, the presented algorithm and epilepsy detection method is verified with data from EEG recordings in a rodent model. We have used the Genetic Absence Epilepsy Rat from Strasbourg (GAERS), which represent one of the best established rodent models for generalized epilepsy. The rats show seizures with characteristic “spike and wave discharge” EEG patterns. For this study, male rats between 6 and 9 months were implanted with epidural electrodes. In total recordings with a length of more than 150 h are available. The data set has been made available via open access on the portal IEEE Dataport [22]. Experiments were performed in accordance with the German law on animal protection and were approved by the Animal Care and Ethics Committee of the University of Kiel.

## 3. Hardware Description

State-of-the-art ultra-low-power microcontrollers allow the execution of complex CNNs complying with real-time, power and size requirements of implantable systems. The microcontroller board chosen for implementing the CNN on hardware is the GAPuino Board developed by GreenWaves Technology [23]. The main processing unit is a GAP8, which is a multi-core RISC-V processor derived from the PULP platform. It is optimized through different approaches to run IoT applications on an ultra-low power base, especially CNNs. These approaches include a powerful programmable parallel processing unit, a hardware convolutional engine and an on-chip power management to reduce the component count while maximizing battery power down-conversion efficiency. The board is an Arduino Uno form factor board including several peripheral interfaces necessary for prototyping [24]. The open source RISC-V-based processor is chosen to enable complete customization and free use of the processor for implantable systems. In future, it is planed to adapt and optimize the RISC-V hardware, especially the hardware convolutional engine, to the specific needs of biosignal processing. In addition, the RISC-V processor architecture is forecasted to be an important processor architecture in industry and research within the next 5 years with over 60 billion processor cores fabricated [25].

## 4. Implementation

### 4.1. Dataset Preparation

Seizure detection can be modeled as a time-series classification problem that classifies the input data into ictal and inter-ictal parts. The CHB-MIT dataset is used as the input for a convolutional neural network. The EEG files of the dataset are preprocessed as described in detail in the Appendix A. The dataset contains a labeling of epileptic activity into ictal and inter-ictal. This labeled EEG data is used for further analysis. The CNN is trained for each patient individually. The datasets of each patients are processed separately and data from individual patients is not shared between the patients, neither in training or test nor in validation phase.

The holdout-method is used to split the dataset into training, validation, and test set. The ratio between these three parts is 60-20-20. The training set is used to train the neural network. On the basis of the validation set, the model optimizes its weights. The final model will be the model which maximizes the classification performance for the validation set. Consequently, the performance on the validation set is not a good estimation for the performance of unseen data. This problem is solved by using the third split: the data set. The data set is only used the test of the final model. It is therefore a good database to evaluate the performance of the model on unseen, new data. The 60-20-20 split is done in the same manner for the ictal data as well as for the inter-ictal data.

For most patients the files contain recordings of 23 channels. However, for almost every patient adjustments have to be conducted to provide a homogeneous input. Empty channel recordings or strongly alternating amplitudes for single channels have been excluded from the analysis.

### 4.2. Data Structuring

The CNN of the epileptic seizure detector processes time signals with a dedicated and fixed length. The dataset is split into parts with a length of 1 s. These blocks of data are the input for the neural network. The length of 1 s is selected to keep the time period of the runtime of a forward pass low while not losing valuable time-dependent information, which is necessary for real-time sensor and actuator systems [12]. The forward pass is the calculation process of traversing through all neurons from first to last layer. The procedure to get these short signal windows is to slide a window with the dimension $W=E\times (T\;\cdot \;f_{s})$ over the data, where *E* are the number of channels, *T* is the time length of the window and $f_{s}$ is the sampling frequency. A sampling frequency of $f_{s}=256\mathrm{Hz}$ and E=23 channels leads to an input matrix of the size 23×256 for T=1 s.

**Figure 1:**
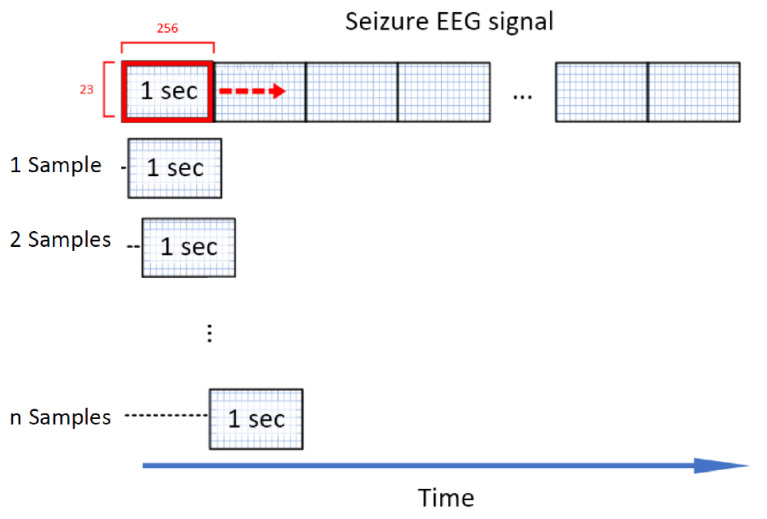
Sliding window technique [27]: A window with a length of 256 samples/1 s is sliding over seizure data with a step size of S=1 sample to generate extra seizures for a balanced training set.

For the inter-ictal data, the window is sliding with no overlap. As already stated, there is an imbalanced number of ictal and inter-ictal data, which is generally challenging for classifiers [26]. Truong et al. [27] propose an approach to solve this problem by generating additional ictal data for training. Similar to the windowing process for the inter-ictal data a window of the dimension 23×256 is shifted over the ictal recordings. The difference here is that the window is only shifted by one sample per iteration, compared to 256 samples for non-ictal data. This corresponds to an overlapping of 99.6%, illustrated in Figure 1. To exemplify this, a epileptic seizure with a length of 10 s is considered. Without the overlapping techniques, this seizure would be cut into chunks of 1 s length generating 10 seizure events. Using the overlapping technique, 2304 seizures events with a respective length of 1 s (256 samples) are generated. This massive overlapping technique is only used for seizure data in the validation and training set. The test set is cut into samples without overlapping.

An EEG signal recorded at the head of a patient is easily corrupted by physiological and non-physiological artifacts such as action potentials from scalp muscle or motion of EEG cables, respectively [20,21]. The CHB-MIT dataset is partially corrupted by such artifacts. The recordings, e.g., contain 60Hz noise, caused by the power supply. This 60Hz noise differs between channels as well as between patients. To reduce the data processing and thus the hardware requirements, a signal preprocessing is not implemented in this work. It is not known to the authors, and not stated in the description of the dataset, if the dataset contains preprocessed data or if the recording equipment of the dataset performs data preprocessing of any kind. For future implantable systems it is assumed that the amount of distortions is reduced due to internal intracranial recordings compared to external recordings.

### 4.3. CNN Architecture

**Figure 2:**
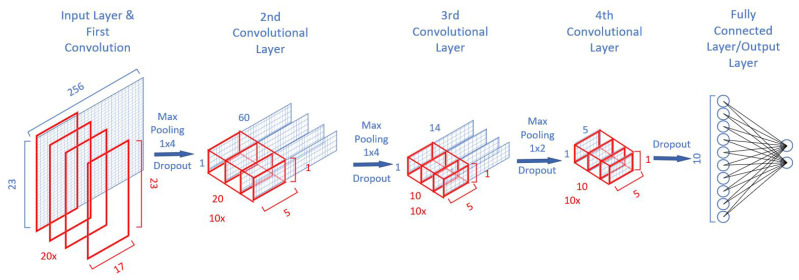
Schematic depiction of the CNN architecture showing the convolutional layers with their respective input matrix (blue rectangles) and kernels (red rectangles). Input is a 23×256 matrix. Between each convolutional layer, a dropout layer and max pooling layer is placed. Output are the two classes ictal and inter-ictal.

The architecture of the CNN used in this work is illustrated in Figure 2 and based on the SeizureNet CNN [12]. In [12], various architecture elements and functions are analyzed in order to evaluate the runtime and memory requirements for an energy-efficient seizure detecting classifier. This includes layer types like convolutions, dense layers (fully connected layer), pooling layers, and different activation functions. While the network in [12] was evaluated using the “Epilepsiae” dataset, especially the intracranial EEG dataset recorded at the University of Freiburg, our work is based on the CHB-MIT [21] dataset. The CNN in our work is optimized for ultra-low-power and energy consumption for future implantable systems. Training, verification and optimization of the CNN developed and presented in our work is performed in MATLAB using the MATLAB deep learning toolbox. The source code has been made available online under open access license [28] to enable rapid adoption in future research projects.

The implemented model architecture for this work is illustrated in Figure 2. The input is a 23×256 matrix. The first layer is a convolutional layer using a kernel with the dimensions 23×17. In the first layer, a convolution over all electrodes is chosen. The data from a multi-channel EEG recording are not spatially uncorrelated and with the selected size the kernel size can be reduced effectively. This approach is similar to spatial pattern recognition approaches and was also recommended in [11]. By adding a filtering over time, the first layer can efficiently learn spatial-time features. For the first layer, the number of kernels is given by 20 × to provide a sufficient quantity of learnable patterns while keeping the amount of weights to be trained on a low level. By implementing a kernel over all electrodes, the output of the first layer is significantly smaller than the input. This is important when taking the needed memory into account. For all convolutional layers the kernel is sliding over the input with a stride of S=1.

The next three convolutional layers extract key features and reduce the dimensionality of the network. The kernel size for the second layer is 10×1×5×20. For the third and fourth layer it is 10×1×5×10. A rectified linear activation function (ReLu) is used for each layer. The output layer is a 10×2 fully connected layer using a sigmoid activation function for each of the ten hidden neurons. As the classification task is to decide between ictal and inter-ictal recordings, each output neuron stands for a class. With a softmax function right at the end of the neural network the probability for the two output classes is calculated.

To reduce the output size for layer 1–2 even further, each convolutional layer is followed by a 1×4 max pooling layer with a stride of S=4. The third convolutional layer is followed by a 1×2 max pooling layer with a stride of S=2. To avoid overfitting, each layer except the output layer also contains a dropout layer with a dropout rate of 20%. The dropout layers are only used during the training phase.

### 4.4. CNN Hardware Implementation

The trained network is implemented on a RISC-V based GAP8 ultra-low-power microprocessor. To make use of its efficiency-increasing hardware convolutional engine and to implement the MATLAB trained CNN on the GAP8, processor optimizations and adjustments have to be made. This includes an adaptation of the network architecture, a transmission of the network architecture from MATLAB to C-Code and a quantization of all parameters including the input matrix. This is necessary, as GAP8 comes, for energy efficiency purposes, without a floating point unit. The quantization performed is an 16-bit Q1.14 fixed point quantization. The quantized parameters on the GAPuino Board are stored in integer form.

In total the CNN has a number of 10.162 trainable parameters and a memory requirement of 62.7kB for 32-bit floats. The detailed structure, the number of trainable parameters and the required memory size per layer are presented in the Appendix A, Table A2.

To implement the CNN trained in MATLAB on the GAPuino Board the TensorFlow SDK used. The Greenwaves GAP8 SDK only supports quadratic convolutions. As the architecture developed in Matlab includes only non-quadratic kernels (23 × 17, 1 × 5 etc.) the number of layers and the filter dimensions have to be changed. The new architecture is given in Table A2. The network only contains two convolutional layers with a kernel size of 5 × 5 with a stride S=1 and a fully connected layer of the size 2440 × 2. The Max Pooling layers are reduced to a 2 × 2 pooling with a stride S=2.

### 4.5. Transferability of the CNN Based Classifier

The transferability of the presented algorithm and epilepsy detection method to classification tasks of the same structure is verified with data from EEG recordings in a rodent model. From the data set, random sample data sets are selected for further analysis. To meet the requirements of rat recordings, the CNN was adopted in such a way that only single channel recordings were used for training. The kernel matrix was adjusted to 1600 instead of 256 to meet the sampling frequency requirement of $f_{s}=1.6\mathrm{kHz}$ of the rats model recording. This ensures that a time frame with a length of 1 s is analyzed and equivalence to the CHB-MIT dataset is maintained. Measurements and results are presented in Section 5.5.

### 4.6. Seizure Prediction Based on Pre-Ictal Data

From a patient’s perspective it would be highly desirable to detect an epileptic seizure before it occurs, instead of a detection during its occurrence. This would allow to issue warnings and take precautionary measures [29]. The ability to predict seizures with the developed CNN-based classification model is analyzed. While no clear unified definition of the length of the pre-ictal phase exists, various works define a period of up to 1 h before a seizure onset as pre-ictal [30,31,32]. In this section, only EEG data from non-seizure files are used for training, validation, and testing, and the models are trained in MATLAB. The seizure recordings are analyzed for different time periods defined as pre-ictal phases for 19 out of the 20 patients. It is assumed that the prediction quality depends on the length of the pre-ictal phase. If information about the upcoming seizure is available in the pre-ictal phase it can be expected that the prediction quality increases when longer time periods of the pre-ictal phase are taken into consideration by the classifier. The length of the pre-ictal phases are selected as 5, 10, 20, and 30 min. The performance of the prediction and the influence of the length of the pre-ictal phase is analyzed.

## 5. Measurements and Results

### 5.1. Evaluation Metrics

The performance of the CNN is evaluated with three metrics quantifying the quality of the binary classification task:

- Sensitivity, also called true positive rate (*TPR*): A measure for the proportion of ictal sequences (positives) that are correctly classified by the model as a seizure.
  (1) $$TPR=\frac{TP}{P};$$
  with *TP*: true positives, i.e., ictal sequences correctly classified as a seizure; *P*: positives, i.e., the total number of ictal sequences (positive cases) in the dataset.
- Specificity, also called true negative rate (*TNR*): Ratio of inter-ictal sequences correctly classified by the model as a non-seizure.
  (2) $$TNR=\frac{TN}{N};$$
  with *TN*: true negatives, i.e., inter-ictal sequences correctly classified as a non-seizure; *N*: negatives, i.e., the total number of inter-ictal sequences (negative cases) in the dataset.
  As the specificity of the classifier is very high with values of of 0.998 and higher, the specificity is measured in units of false positive rate for better comparability with other works.
- False positive rate (*FPR*) per hour (fp/h): Number of inter-ictal sequences (with a length of 1 s) wrongly classified by the model as a seizure per hour. The relation between these measures is given by:
  (3) TNR=1−FPR.
- AUC-score: Area under receiver operating characteristic (ROC) Curve—Measure of the model’s ability to distinguish between the seizure and non-seizure classes.

These metrics ensure a comparability between different patients and results from related work.

### 5.2. MATLAB Classification Results

For each patient, an individual CNN is trained based on a personal dataset. The training phase of the neural network is limited to a length of 25 epochs. This makes the results comparable and provides equal conditions for all patients. All together the training is performed for 20 patients. All evaluation metrics are calculated for each patient separately.

**Figure 3:**
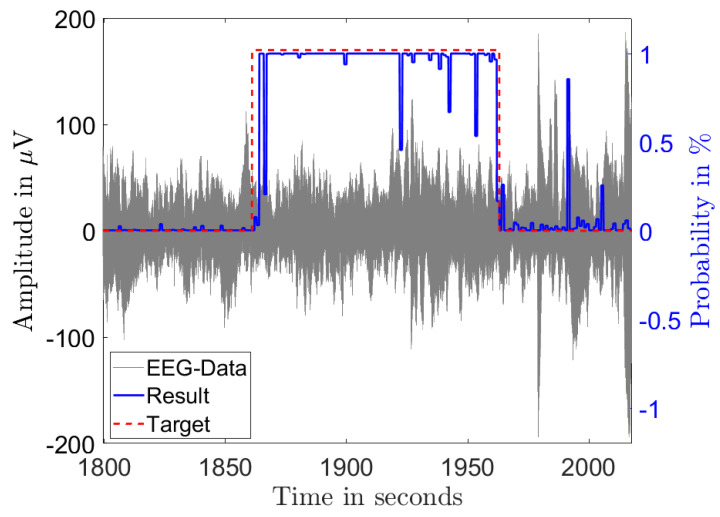
Single channel EEG data (gray) from a seizure record file of patient 1 showing 101 s of diagnosed seizure (red) with the output probability of the classification (blue).

**Figure 4:**
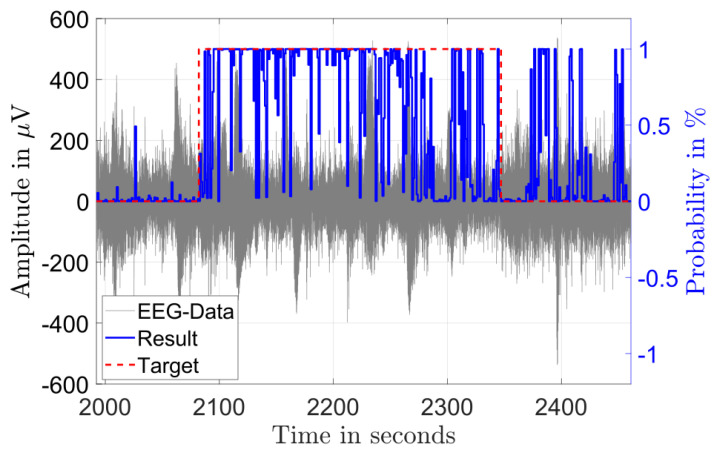
Single channel EEG data (gray) from a seizure record file of patient 8 showing 264 s of diagnosed seizure (red) and the output probability of the classification (blue).

Figure 3 and Figure 4 illustrate the results of the classification in the time domain. The figures show the amplitude of a single channel EEG data in gray and the classification result as a probability in blue. A classified seizure event with a length of 101 s (marked in red) with a high detection probability throughout the event and low probability outside of the event is depicted in Figure 3. Figure 4 shows a classified seizure event with a length of 264 s (marked in red) and a highly fluctuating detection probability (blue). A possible reason for the poor detection probability in the event of Figure 4 could be the high background and low SNR in the signal (grey).

**Figure 5:**
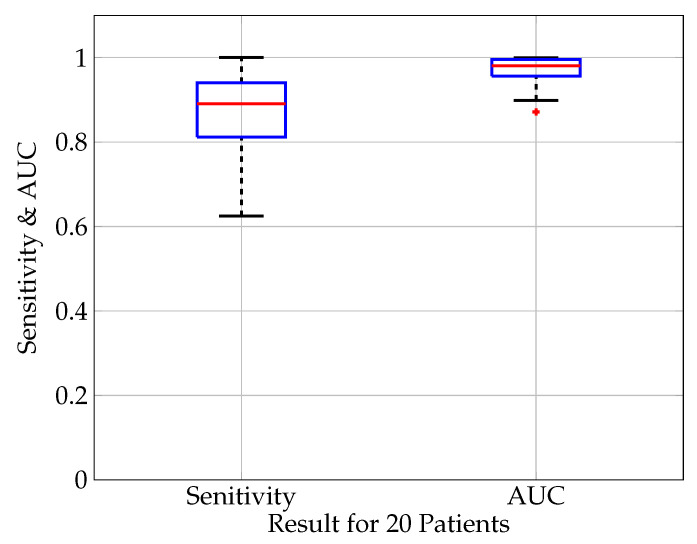
Boxplot (median value (red), lower and upper quartile (blue), min. and max. value (black), outlier (red cross)) of the evaluation measures sensitivity and AUC score for 20 patients. The median sensitivity is 0.90, 75 percentile: 0.94, 25 percentile: 0.81. The median AUC score is 0.98, 75 percentile: 0.99, 25 percentile: 0.98.

The detection sensitivity of all analyzed patients is shown in Figure 5 for a classification threshold of 0.5. The median sensitivity is 90% with a minimum outlier of 62.5% and a maximum of 100%. In this and the subsequent analysis, the median value instead of the average value is calculated in order to take into account possible outliers due to bad signal quality or corrupted (non-physiological) data in single patient’s data sets.

The AUC-score is illustrated in Figure 5 showing a high median of 98% with the high quartile reaching 90% and the low quartile 95%. Overall, the sensitivity is the most important metric to evaluate the detection algorithm. Its importance is related to the fact that not detecting a seizure is worse than having a false positive alarm. The AUC is mainly an additional score to compare different neural network architectures since it is independent of the classifier threshold.

**Figure 6:**
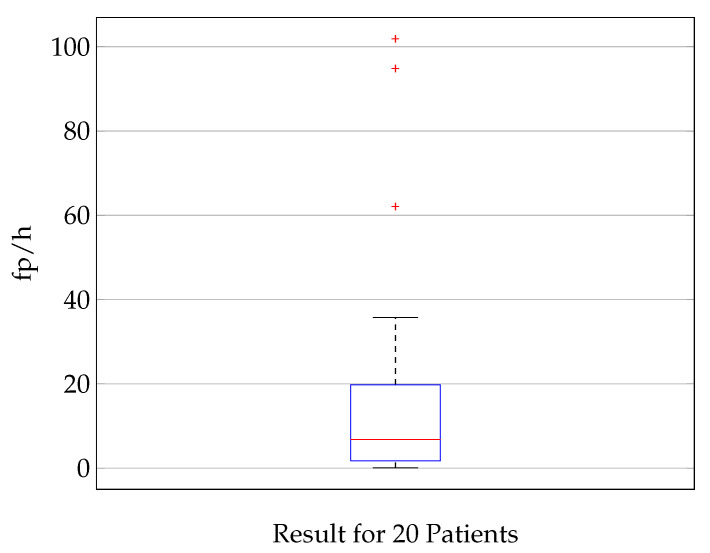
Illustration of the specificity of the classification showing a boxplot (median value (red), lower and upper quartile (blue), min. and max. value (black), outlier (red cross)) of the fp/h for 20 patients. The median fp/h is 6.8, 75 percentile: 19.8, 25 percentile: 1.75. The analysis is done on time signals with a length of 1 s. A false positive rate of 6.8 fp/h corresponds to a specificity of 0.998, this means that 99.8% of inter-ictal time frames of 1 s are classified correctly.

The distribution of the false positives per hour, which is according to Equation (3) a measure for the specificity, is shown in Figure 6. The median fp/h rate is 6.8 fp/h. Seventy-five percent of the results show less than 20 false positives per hour. For three patients, the fp/h rate is significantly higher (101, 95, and 65) than it is for the other patients. It is hypothesized and verified with a random sample test that this is due to a lower signal quality and higher noise level for these three patients. The best 5 patients stand out with a maximum of 1.7 fp/h. The minimum is 0.5 fp/h for patient 2.

### 5.3. Classification Results for Hardware-Optimized CNN

**Figure 7:**
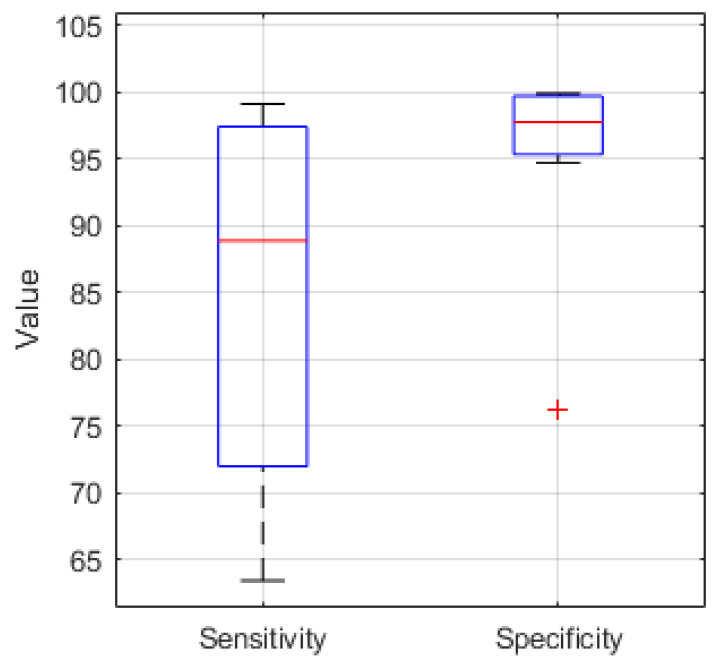
Sensitivity and Specificity boxplot (median value (red), lower and upper quartile (blue), min. and max. value (black), outlier (red cross)) for 10 EEG recordings classified in Python with a median sensitivity and specificity of 88.8% and 97.7%, respectively.

The sensitivity and specificity for 10 EEG recordings classified in Python using the hardware optimized CNN structure are presented in Figure 7. The median sensitivity is 88.8%, the median specificity is 97.7%.

**Figure 8:**
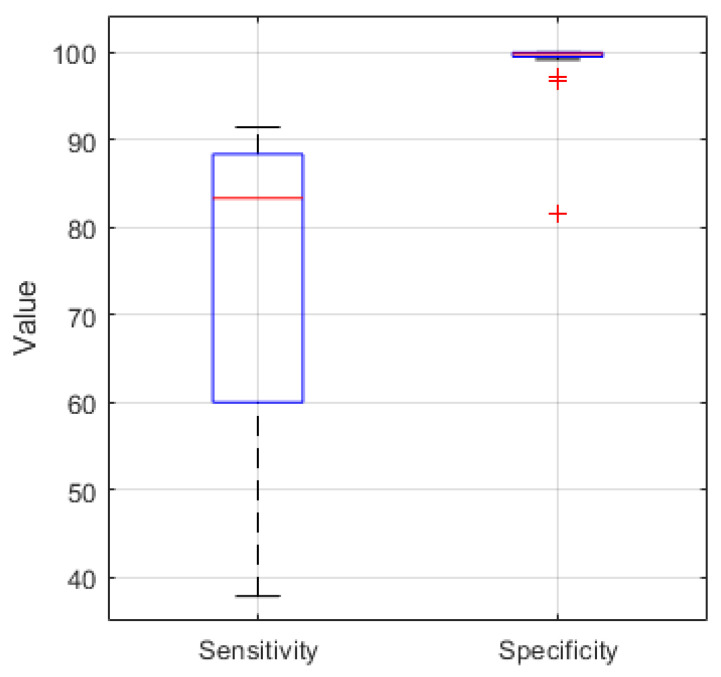
Sensitivity and Specificity boxplot (median value (red), lower and upper quartile (blue), min. and max. value (black), outlier (red cross)) for 21 EEG recordings classified in MATLAB with a median of 83.3% and 99.8%, respectively.

For comparison Figure 8 shows the boxplot of the sensitivity and specificity of the EEG recordings classified in MATLAB. For the 21 analyzed recordings the median values are 83.3% and 99.8% respectively.

### 5.4. Power Consumption

**Figure A1:**
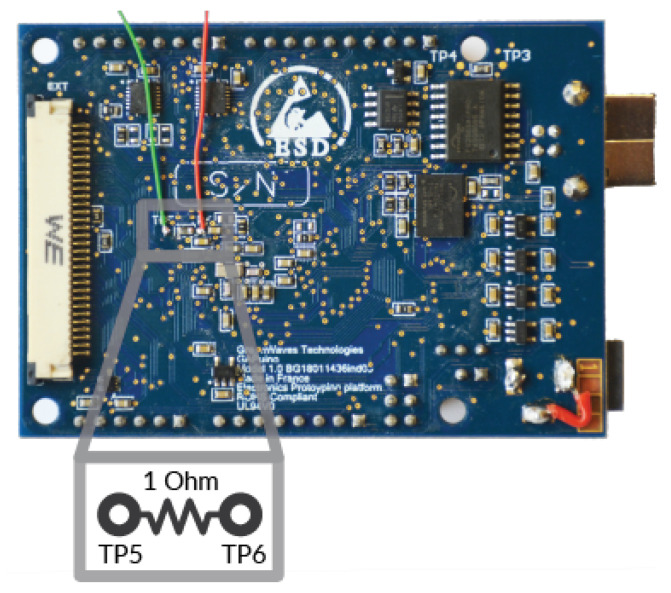
Measurement setup for power consumption showing a R=1Ω shunt resistor at the bottom of the of the GAPuino evaluation [23] board with two measurement points (TP5 and TP6).

The power consumption of the classification task is measured using a shunt resistor (R=1Ω) connected in series with the power supply of the processor of the GAPuino Board. While the processor performs the classification task, the voltage drop at the shunt resistor is measured. Based on the voltage and the value of the resistor, the power consumption is calculated. The measurement setup is depicted in Figure A1.

**Figure 9:**
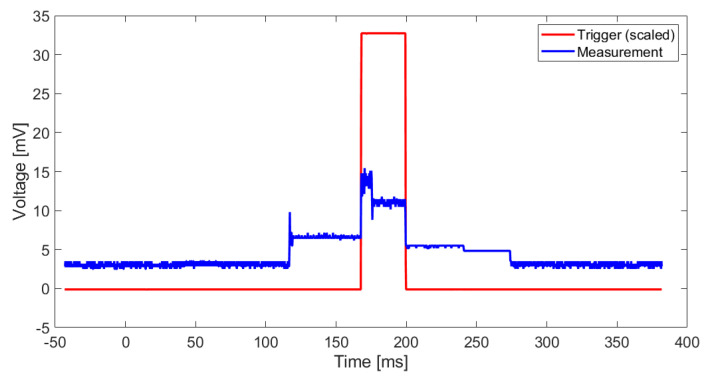
Measured voltage between TP5 and TP6 to measure the power consumption of GAP8 while classifying 1 s of EEG data (blue), trigger signal indicating the start and the end of the processing of 1 s of EEG data.

In order to have a trigger for the energy measurement, a digital I/O port is set to high before the GAP8 kernels are launched and set to low after the classification task is done. The voltage curve while classifying 1 s of EEG data is shown in Figure 9. The time period between on- and offset of the 8 processor kernels is marked by the trigger. The classification task of 1 s of EEG data takes a length of 33.5ms. The maximum voltage reached is 15.44mV. The trigger is scaled by a factor of 100 to simplify the representation. The maximum power consumption is 238.4 μW. The average consumption over the classification period is 140 μW. The energy required for one classification task is E=4.9 μJ. The declared energy consumption is for classifying 1 s of EEG data and only for the GAP8 processor itself. The consumption of the whole board is not measured.

### 5.5. Verification with EEG Recordings in a Rodent Model

**Figure 10:**
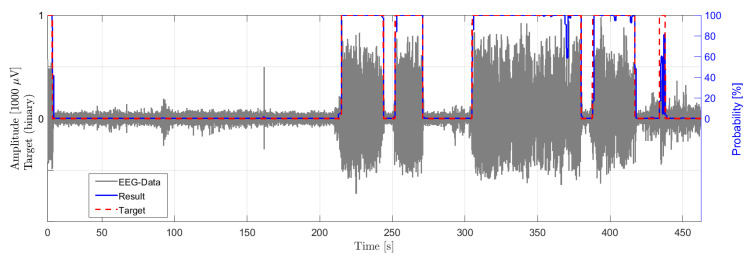
Single-channel recordings from a GAERS rat (gray) with a duration of 470 s showing a seizure event (red), as diagnosed by an expert, and the output probability of the classification (blue).

The presented algorithm and epilepsy detection method are verified with data from EEG recordings in a rodent model. Figure 10 shows one channel of the time signal of a recording of an epileptic rat with seizures and the classification result of the algorithm. The gray signal shows the recorded EEG signal in μV. The dashed red line shows the classification target indicating a seizure event with target value one and a non-seizure event with target value low, classified by neurological experts. The blue solid line shows the output probability of the classifier. As shown in the example in Figure 10, the four seizure events are detected by the classifier and the classification results of the CNN match very well with the classification of experts.

This exemplified result illustrates, that the developed CNN based seizure detection model is generally applicable and transferable to similarly structured classifications task. The performance of the adoption of the CNN is not evaluated in this work.

### 5.6. Seizure Prediction

**Figure 11:**
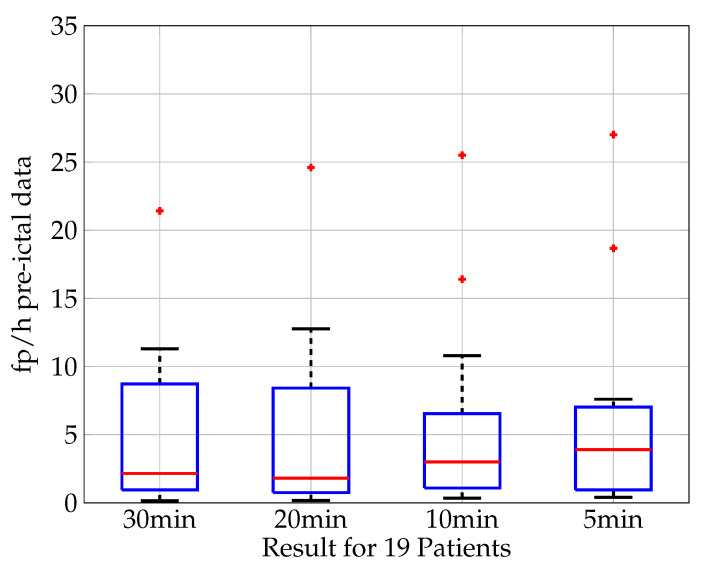
Seizure prediction based on pre-ictal data. Boxplot (median value (red), lower and upper quartile (blue), min. and max. value (black), outlier (red cross)) of the classification results in fp/h for 19 patients. The time period defined as “pre-ictal” varies from 30, 20, 10 to 5 min. The classification results show a median false positive rate of 2.15 fp/h for a pre-ictal time period of 30 min, of 1.8 fp/h for 20 min, 3.0 fp/h for 10 min and 3.9 fp/h for 5 min.

For 19 out of the 20 patients, the seizure recordings are analyzed for different time periods defined as pre-ictal phases prior to seizure onset. Figure 11 shows the result of the analysis in the boxplot of fp/h for pre-ictal time periods. For this work the analysis is conducted for the time periods of 30, 20, 10, and 5 min. The classification results show a median false positive rate of 2.15 fp/h for a pre-ictal time period of 30 min, of 1.8 fp/h for 20 min, 3.0 fp/h for 10 min, and 3.9 fp/h for 5 min. While the shortest phase of 5 min shows the worst prediction, a clear trend for an increase in prediction quality for longer periods of pre-ictal phases can be seen. This strengthens the assumption that information about the upcoming seizure is available in the pre-ictal phase and that the prediction quality increases when more information is available or longer time periods are taken into consideration, respectively.

## 6. Comparison with State-of-the-Art

A comparison of the overall classification performance of the developed approach with recently published work is shown in Table 1. This comparison contains recent works that focus on future implantable systems. Thus, the approaches focus on low hardware complexity and low power requirement. Although it has to be admitted that it is difficult to compare the performance of classifiers based on classification results from different databases, it can be stated that all three classifiers show compatible performance.

A comparison of the power consumption with measurement results from state-of-the-art solutions is shown in Table 2. The average power consumption for a classification is more than 80% smaller than in [12]. With respect to energy consumption the presented work achieves a reduction by a factor of 6.9 and higher compared to that in [27], which consumes 34–90 μJ for each classification.

The performance of different algorithms to detect epileptic seizures is compared in Table 3, based on the work in [33]. In this comparison, hardware requirements are not considered. The analysis are based on different data sets, thus the performance parameters cannot be compared directly. Nevertheless, it can be stated that it is possible to achieve comparable sensitivity and specificity with a variety of algorithms.

## 7. Conclusions

In this work, an epileptic seizure detection algorithm using a convolutional neural network has been presented, analyzed in MATLAB and implemented on an ultra-low-power RISC-V processor. In an implementation of the CNN on a RISC-V-based GAPuino microcontroller, a sensitivity of 85% is reached. The classification of 1 s of EEG data requires E=4.9 μJ, which is suitable for low-power implantable systems. The specificity is higher than 99%. The classification of 1 s of input data takes 35 ms. Thus the low latency required for real-time applications is achieved. The proposed detector reduces the power consumption by the factor of 6 compared to related approaches. This is reached by the adoption of the CNN and by exploiting the hardware convolution engine of the of GAP8 microprocessor, which allows an energy efficient computation of the convolution operator. The CNN presented here is trained individually for each patient. Accordingly, this approach is not limited to a specific type of epilepsy. Instead it is generally applicable for epilepsy with recurring and comparable seizure events. This was confirmed with recordings from a rat model.

The classifiers, codes, and the data from recordings in the rodent model are made available to the public under open access license. This enables easy reuse and rapid adoption of the presented approach for future developments and applications.

## Figures and Tables

**Table 1:** Comparison of the overall classification performance of the developed approach with published work.

	SeizureNet [12]	IntegerNet [27]	This Work
Database	iEEG dataset	CHB-MIT	CHB-MIT
Median sensitivity	96%	unspecified	90%
Median False positives per hour	10 fp/h	unspecified	6.8 fp/h
AUC-score	93%	94%	98%

**Table 2:** Comparison of the energy consumption of a classification task for different chips and classifiers while running a seizure classification.

	SeizureNet [12]	IntegerNet [27]	This Work
Chip	unspecified	Microcontroller in 45 nm, 0.9 V CMOS process	GAP8 (8 core, RISC-V)
Power	850 μW	unspecified	140 μW
Energy	unspecified	34–90 μJ/classification	4.9 μJ/classification

**Table 3:** Comparison of the performance of different algorithms for seizure detection independent of the hardware requirements, based on those in [33].

	Gabor [34]	Kelly [35]	Hopfengärtner [36]	This Work
Algorithm	Neural networks, CNET	Pattern- match regularity statistics, local max. frequency, amplitude variation	Power spectral analytical techniques, Short time Fourier transform	Convolutional Neural Network
EEG Sample [h]	528	1200	3248	865
Patients	22	55	19	20
Seizures	62	146	148	198
Sensitivity	90.3	79.5	90.9	90
Specificity [fp/h]	0.71	0.08	0.29	6.8

## Data Availability

The GAERS data set is available via open access on the portal IEEE Dataport [22]. The MATLAB source code is available online under open access license [28] to enable rapid adoption in future research projects.

## Appendix A

The Appendix A gives detailed information on the dataset, implementation, and the code in Matlab and TensorFlow.

### Appendix A.1. Dataset

In the majority of cases the dataset is segmented in one-hour files, where files with a seizure occurring are called ’seizure records’ and files without a seizure ’non-seizure records’. The recordings are formatted in the European Data Format (.edf). The information about used channels as well as starting and ending of a seizure period is stored in a separate text document.

### Appendix A.2. Hardware Implementation

#### Appendix A.2.1. Dataset Preparation

The recordings of patients 11, 14, 19, 20, 21, and 22 contain empty channels. As these do not represent physiological data, they are not considered in the model. For patients 12, 13, and 18, the assigned scalp electrodes for each channel change massively throughout the recorded files. For this reason, the EEG data of these three patients is discarded from the analysis. The recordings of patient 17 contains only 22 channels, this patient is neglected too.

#### Appendix A.2.2. CNN Architecture

The Matlab code created for implementing the CNN in this work has been made available at Github [28]. The folder CHB_MIT includes all used functions, for data import of EEG data data preprocessing (e.g., removal of empty channels). The main function for constructing the CNN is located in the file CHB_MIT\seizure_detection_cnn\seizure_nn.m The folder Basis includes basic Matlab functionalities and auxiliary functions, e.g., functions for reading of data format EDF or storage of Matlab matrices in numpy arrays.

#### Appendix A.2.3. CNN Hardware Implementation

The architecture of the CNN, its dimensions and the number of filters is summarized in Table A1.

**Table A1:** Structure of the CNN, dimensions, and number of filters.

Layer	Name	Dimension	Number of Filters
	Input	23×256×1	-
1	Conv2D	23×17	20
	MaxPool2D (1×4)		
	Dropout (0.2)		
2	Conv2D	1×5	10
	MaxPool2D (1×4)		
	Dropout (0.2)		
3	Conv2D	1×5	10
	MaxPool2D (1×2)		
	Dropout (0.2)		
4	Conv2D	1×5	10
	Dropout (0.2)		
5	Conv2D	1×1	1
6	Sigmoid	-	-

#### Appendix A.2.4. CNN Implementation in Tensorflow

The structure of the CNN is adopted and optimized to meet the requirements of the GAPuino board using the GAP8 software development kit. The detailed structure and the required memory size per layer is presented in Table A2.

**Table A2:** Structural adaptation of the CNN for implementation on GAPuino Board using the GAP8 SDK and memory size per layer. In total the CNN has a number of 10.162 trainable parameters and a memory requirement of 62.7 kB for 32-bit floats.

n-Layer	Operation	Output	Size
1	Input (23 × 256)	23 × 256	5888
2	10 × Convolution (5 × 5)	19 × 252 × 10	260
3	MaxPooling (2 × 2)	9 × 126 × 20	-
4	Dropout (0.2)	-	-
5	20 × Convolution (5 × 5)	5 × 122 × 20	5020
6	MaxPooling (2 × 2)	2 × 61 × 20	-
7	Dropout (0.2)	-	-
8	FullyConnected (2440 × 2)	2	4880
9	Softmax (2)	2	2
**Total Number of trainable parameters**		**=**	**10,162**
**Input (23 × 256)**		**=**	**5888**
**Needed memory for 32-bit floats**		**=**	**≈62.7 kB**

### Appendix A.3. Measurements and Results

#### Power Consumption

The measurement setup for the power consumption during classification is depicted in Figure A1. The shunt resistor and the test points TP5 and TP6 are depicted at the bottom of the board.

## References

[1] World Health Organization (2019). Epilepsy: Key Facts. https://www.who.int/news-room/fact-sheets/detail/epilepsy.

[2] Bishop P., Allen C. (2003). The impact of epilepsy on quality of life: A qualitative analysis. Epilepsy Behav..

[3] Sperling M.R. (2004). The Consequences of Uncontrolled Epilepsy. CNS Spectrums.

[4] National Institute of Neurological Disorders and Stroke (2015). Epilepsy: Hope Through Research.

[5] Löscher W., Potschka H., Sisodiya S.M., Vezzani A. (2020). Drug Resistance in Epilepsy: Clinical Impact, Potential Mechanisms, and New Innovative Treatment Options. Pharmacol. Rev..

[6] Chen Z., Brodie M.J., Liew D., Kwan P. (2018). Treatment Outcomes in Patients With Newly Diagnosed Epilepsy Treated With Established and New Antiepileptic Drugs: A 30-Year Longitudinal Cohort Study. JAMA Neurol..

[7] Gernert M., Feja M. (2020). Bypassing the Blood–Brain Barrier: Direct Intracranial Drug Delivery in Epilepsies. Pharmaceutics.

[8] Trinka E., Boon P., Mertens C. (2018). Neurostimulation for drug-resistant epilepsy: A systematic review of clinical evidence for efficacy, safety, contraindications and predictors for response. Curr. Opin. Neurol..

[9] Astrakas L., Konitsiotis S., Tzaphlidou M., Stevanovic D. (2012). Automated Epileptic Seizure Detection Methods: A Review Study. Epilepsy—Histological, Electroencephalographic and Psychological Aspects.

[10] Baldassano S.N., Brinkmann B.H., Ung H., Blevins T., Conrad E.C., Leyde K., Cook M.J., Khambhati A.N., Wagenaar J.B., Worrell G.A. (2017). Crowdsourcing seizure detection: Algorithm development and validation on human implanted device recordings. Brain A J. Neurol..

[11] Lawhern V.J., Solon A.J., Waytowich N.R., Gordon S.M., Hung C.P., Lance B.J. (2016). EEGNet: A Compact Convolutional Network for EEG-based Brain-Computer Interfaces. J. Neural Eng..

[12] Hügle M., Heller S., Watter M., Blum M., Manzouri F., Dümpelmann M., Schulze-Bonhage A., Woias P., Boedecker J. (2018). Early Seizure Detection with an Energy-Efficient Convolutional Neural Network on an Implantable Microcontroller. arXiv.

[13] Daoud H., Bayoumi M.A. (2019). Efficient Epileptic Seizure Prediction Based on Deep Learning. IEEE Trans. Biomed. Circuits Syst..

[14] Roy Y., Banville H., Albuquerque I., Gramfort A., Falk T.H., Faubert J. (2019). Deep learning-based electroencephalography analysis: A systematic review. J. Neural Eng..

[15] Bahr A., Saleh L.A., Hinsch R., Schroeder D., Isbrandt D., Krautschneider W.H. Small area, low power neural recording integrated circuit in 130 nm CMOS technology for small mammalians. Proceedings of the 2016 28th International Conference on Microelectronics (ICM).

[16] Bahr A. (2017). Aufnahme Von Hirnsignalen Mit Extrem Miniaturisierten Elektronischen Systemen Zur Untersuchung Von Lokalen Neuronalen Vernetzungen.

[17] Thomas S.J., Harrison R.R., Leonardo A., Reynolds M.S. (2012). A Battery-Free Multichannel Digital Neural/EMG Telemetry System for Flying Insects. IEEE Trans. Biomed. Circuits Syst..

[18] Laiwalla F., Lee J., Lee A., Mok E., Leung V., Shellhammer S., Song Y., Larson L., Nurmikko A. A Distributed Wireless Network of Implantable Sub-mm Cortical Microstimulators for Brain-Computer Interfaces. Proceedings of the 2019 41st Annual International Conference of the IEEE Engineering in Medicine and Biology Society (EMBC).

[19] Benabid A.L., Costecalde T., Eliseyev A., Charvet G., Verney A., Karakas S., Foerster M., Lambert A., Morinière B., Abroug N. (2019). An exoskeleton controlled by an epidural wireless brain–machine interface in a tetraplegic patient: A proof-of-concept demonstration. Lancet Neurol..

[20] Shoeb A. (2009). Application of Machine Learning to Epileptic Seizure Onset Detection and Treatment. Ph.D. Thesis.

[21] Shoeb A., Guttag J. Application of Machine Learning to Epileptic Seizure Detection. Proceedings of the 27th International Conference on International Conference on Machine Learning, ICML’10.

[22] Dataport I. (2020). EEG of Genetic Absence Epilepsy Rats (GAERS). https://ieee-dataport.org/open-access/eeg-genetic-absence-epilepsy-rats-gaers.

[23] Greenwaves Technologies (2018). GAP8 Manual. https://greenwaves-technologies.com/manuals/BUILD/HOME/html/index.html.

[24] Flamand E., Rossi D., Conti F., Loi I., Pullini A., Rotenberg F., Benini L. GAP-8: A RISC-V SoC for AI at the Edge of the IoT. Proceedings of the 2018 IEEE 29th International Conference on Application-specific Systems, Architectures and Processors (ASAP).

[25] SEMICO Research Corporation (2019). RISC-V Market Analysis: The New Kid on the Block.

[26] Branco P., Torgo L., Ribeiro R.P. (2016). A Survey of Predictive Modeling on Imbalanced Domains. ACM Comput. Surv..

[27] Truong N.D., Nguyen A.D., Kuhlmann L., Bonyadi M.R., Yang J., Ippolito S., Kavehei O. (2018). Integer Convolutional Neural Network for Seizure Detection. IEEE J. Emerg. Sel. Top. Circuits Syst..

[28] Github (2020). Matlab Source Code. https://github.com/cau-etit-sse/cnn-chb-mit.

[29] Cook M.J. (2021). Advancing seizure forecasting from cyclical activity data. Lancet Neurol..

[30] Eberlein M., Hildebrand R., Tetzlaff R., Hoffmann N., Kuhlmann L., Brinkmann B., Muller J. Convolutional Neural Networks for Epileptic Seizure Prediction. Proceedings of the 2018 IEEE International Conference on Bioinformatics and Biomedicine (BIBM).

[31] Gagliano L., Bou Assi E., Nguyen D.K., Sawan M. (2019). Bispectrum and Recurrent Neural Networks: Improved Classification of Interictal and Preictal States. Sci. Rep..

[32] Ozcan A.R., Erturk S. (2019). Seizure Prediction in Scalp EEG Using 3D Convolutional Neural Networks With an Image-Based Approach. IEEE Trans. Neural Syst. Rehabil. Eng..

[33] Baumgartner C., Koren J.P. (2018). Seizure detection using scalp-EEG. Epilepsia.

[34] Gabor A., Leach R., Dowla F. (1996). Automated seizure detection using a self-organizing neural network. Electroencephalogr. Clin. Neurophysiol..

[35] Kelly K., Shiau D., Kern R., Chien J., Yang M., Yandora K., Valeriano J., Halford J., Sackellares J. (2010). Assessment of a scalp EEG-based automated seizure detection system. Clin. Neurophysiol..

[36] Hopfengärtner R., Kerling F., Bauer V., Stefan H. (2007). An efficient, robust and fast method for the offline detection of epileptic seizures in long-term scalp EEG recordings. Clin. Neurophysiol..

